# A Wireless High-Sensitivity Fetal Heart Sound Monitoring System

**DOI:** 10.3390/s21010193

**Published:** 2020-12-30

**Authors:** Jianjun Wei, Zhenyuan Wang, Xinpeng Xing

**Affiliations:** 1 School of Telecommunications Engineering, Xidian University, Xi’an 710072, China; walter_yu@163.com; 2 Shenzhen International Graduate School, Tsinghua University, Shenzhen 518055, China; xing.xinpeng@sz.tsinghua.edu.cn

**Keywords:** fetal heart sound, fetal heart rate, PVDF piezoelectric film, automatic weight, weighted-index average

## Abstract

In certain cases, the condition of the fetus can be revealed by the fetal heart sound. However, when the sound is detected, it is mixed with noise from the external environment as well as internal disturbances. Our exclusive sensor, which was constructed of copper with an enclosed cavity, was designed to prevent external noise. In the sensor, a polyvinylidene fluoride (PVDF) piezoelectric film, with a frequency range covering that of the fetal heart sound, was adopted to convert the sound into an electrical signal. The adaptive support vector regression (SVR) algorithm was proposed to reduce internal disturbance. The weighted-index average algorithm with deviation correction was proposed to calculate the fetal heart rate. The fetal heart sound data were weighted automatically in the window and the weight was modified with an exponent between windows. The experiments show that the adaptive SVR algorithm was superior to empirical mode decomposition (EMD), the self-adaptive least square method (LSM), and wavelet transform. The weighted-index average algorithm weakens fetal heart rate jumps and the results are consistent with reality.

## 1. Introduction

The condition of the fetus in utero can be monitored effectively with the utility of fetal heart sound. It can be used to judge whether the intrauterine fetus is hypoxic and to reflect the condition of the fetal central nervous system, amongst other things. Meanwhile, the data can be sent to an obstetrician. In this way, contact between the obstetrician and the gravida is significantly increased, allowing potential problems to be found early, and reducing the incidence of fetal mortality. Fetal heart sound acquisition methods can be divided into active and passive forms. On the basis of the Doppler principle and dynamic weighting matrices, Hamelmann improved the signal-to-noise ratio effectively and was able to obtain the fetal heart rate [1]. Khandoker obtained more information related to fetal heart sound based on the same principle [2]. The active method transmits ultrasonic signals to the abdomen of the pregnant woman and changes the fetal environment. Hiroyuki Sato designed a fetal heart sound sensor whose size is about 19.2 cm^3^ [3]. An enhanced non-intrusive monitoring method was proposed by Andreotti and the accuracy of fetal heart rate monitoring was increased [4]. Through these acquisition methods, the gained signal is close to the condition in the uterus. However, noise and disturbance still exist and significantly deteriorate the signal quality. A phonography-based measurement of the fetus was proposed by Kovacs. Segment structures with frequency splitting were used to distinguish the sound signal from the disturbance signals, such as those caused by hiccups, the body’s rotation, and limb movements. However, the sound signal can only reflect the fetal breathing movement and not the condition of the fetal heart [5]. The contact force of the sensor affects the quality of the acquired fetal heart sounds and there exists an optimum contact force for the sensors [6]. During recording, the high-quality segments can be automatically identified and selected, but the interference from the environment remains [7]. The aim of this study was to acquire a high-quality fetal heart signal and obtain the accurate fetal heart rate.

## 2. Structure of the Fetal Heart Sound Monitoring System

**Figure 1:**
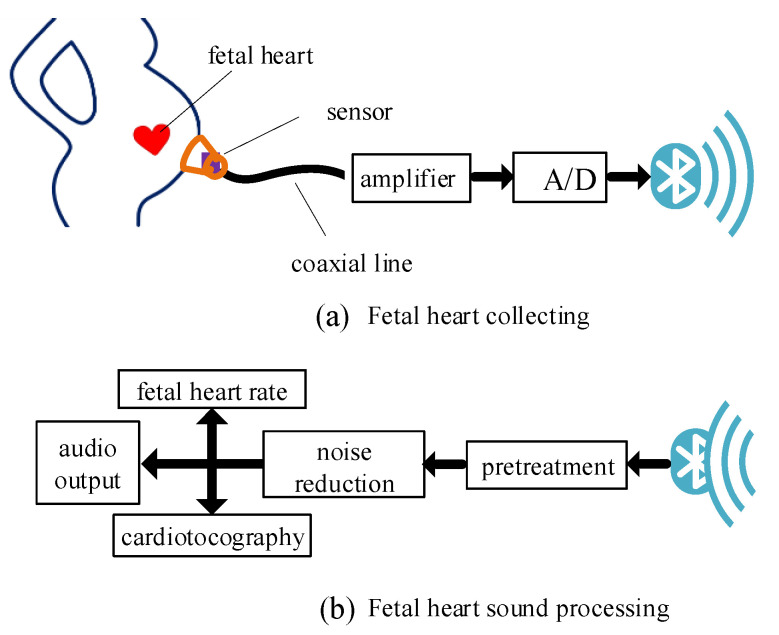
Structure of the fetal heart sound monitoring system.

The wireless fetal heart sound monitoring system is composed of two parts: fetal heart sound collection and processing. Fetal heart sound collection requires a fetal heart sound sensor and a collection module. Fetal heart sound processing includes de-noising of the fetal heart sound and extraction of the fetal heart rate. The whole structure is shown in Figure 1. Figure 1a shows fetal heart sound collection and Figure 1b shows fetal heart sound processing at the terminal.

In the system, the fetal heart sound is converted into an electrical signal by the exclusive high-sensitivity sensor. Then, the weak electrical signal is amplified and converted into a digital signal and transmitted to the terminal via Bluetooth. When the signal arrives, it is processed through data conversion, normalization, mean removal, and so on. The original data are converted to double-precision floating-point operands for the subsequent operations. The application of zero-mean and normalization speed up the algorithm so that the time demands of real-world scenarios are met. The noise is reduced after pretreatment, the fetal heart sound is extracted, and the fetal heart rate is calculated.

## 3. Fetal Heart Sound Collection

The disturbance to the fetal heart sound comes from two factors. The first is the interference from the pregnant woman, such as heartbeats, breathing, rhythmic stomach movements, and the echo of the fetal heart sound on the internal wall. The second is the external disturbance, such as random noise and power-line interference. The body interference is mixed in before the signal reaches the sensor and must be reduced using software at the terminal. External disturbance can be reduced to a minimum by using a proper sensor.

### 3.1. Fetal Heart Sound Sensor

The frequency of fetal heart sound is 10–400 Hz, and this includes components with frequencies lower than 20 Hz. The low-frequency limitation of the traditional microphone is 20 Hz, so the integrity of the fetal heart sound cannot be guaranteed in this way. Polyvinylidene fluoride (PVDF) piezoelectric film can convert sound into electrical signals. The material has a wide frequency response, i.e., 0–400 MHz, and can gather more low-frequency information. The impedance of PVDF is close to that of the human body and the signal reflection is small when they are contacted. Furthermore, the material is thin, light, soft, chemically stable, and it can be cut arbitrarily. Fabrication is simple and the cost is low.

The key to the amplifier connected with the sensor is the connection of the amplifier with PVDF piezoelectric film. When the ambient air vibrates, the film vibrates. The variation in charge between the top and bottom of the film produces an electrical signal. The charge between the top and bottom is the same with a reverse polarization when the film voltage acts as a reference. For this reason, the signal was put into the input of the differential amplifier. The common noise in the two paths was cancelled after difference amplification due to the symmetry of the original signal. The ports of the transmission lines connect the surface and the bottom of the film; the other ports connect the positive and negative terminals of the differential amplifier. Welding cannot be used because the film becomes deformed at very high temperatures, and can even degrade completely. Therefore, a perforation method is preferred. The ground of the circuit is connected to the copper shell of the sensor and the external disturbance is shielded, especially to EMC in the environment. The antenna effect occurs when the normal conductor line is used to connect the module and the disturbance with fixed frequency injections. The coaxial lines are used between the film and the differential amplifier. The collected fetal heart sound signal is transmitted through the wire in the coaxial lines, while a woven wire cloth is connected with the ground to shield it from disturbance.

**Figure 2:**
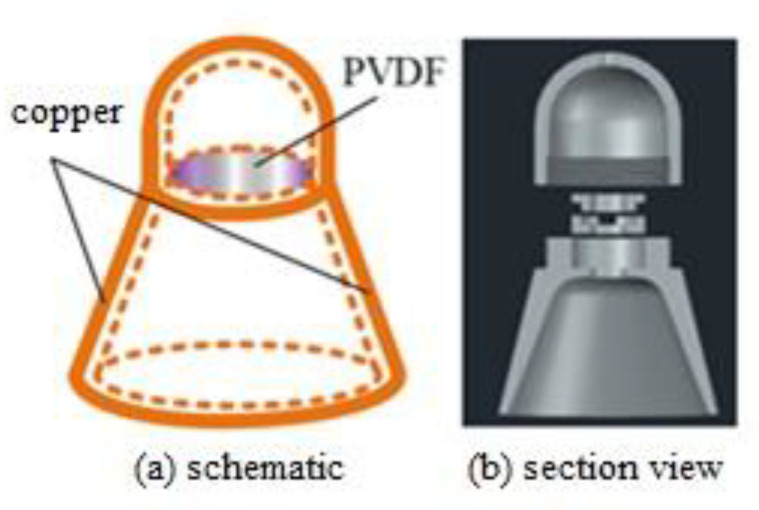
Fetal heart sound sensor.

The fetal heart sound sensor was designed according to the principle of a stethoscope and aerodynamics. It was used in the closed environment and is showed in Figure 2.

Figure 2a presents the principle of the sensor and the position of the film. Figure 2b is a 3D profile. The film was fixed by two clips in the center of the sensor and the conductor lines were drawn.

### 3.2. Fetal Heart Sound Collection Module

**Figure 3:**
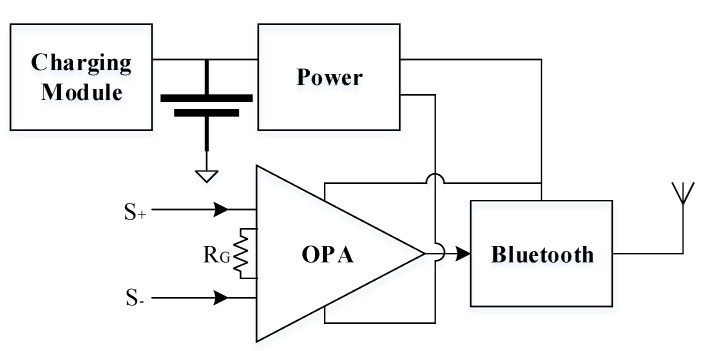
The structure of fetal heart sound collection module.

The original signal collected by the sensor is processed by the collection module in the fetal heart sound monitoring system. The module includes an amplifier, Bluetooth, a charging circuit, and a feed circuit. They are shown in Figure 3.

The amplification multiple is controlled by the feedback resistor, *R_G_*, in the amplifier. The relationship between resistance and amplification multiple is:

(1) $$G=\frac{49.4\;k\Omega }{R_{G}}+1$$

The amplification multiple was 12 after the experiment; it corresponds to a resistance of 4.7 k.

The sensor in contact with the skin has zero potential just like the reference with the differential amplifier, because the positive and negative terminals of the amplifier are connected with the top and bottom surfaces of the PVDF. Bioelectricity exists in the skin and disturbs the collected sound. Through the adoption of this structure, the disturbance induced by human bioelectricity was reduced. The fetal heart sound is sent to a terminal, such as a smart phone, via Bluetooth, after amplification. The smart phone is the main communication technology in daily life and almost all have Bluetooth capabilities, making this relevant for real-world applications. The Bluetooth connection-oriented approach, Santa Cruz Operation (SCO), was adopted and the data were transmitted in real time from the collection module to the smart phone. The rechargeable lithium battery was charged using a charging circuit when it is running low. The positive and negative voltage sources, which were produced by the feed circuit, supplied Bluetooth and the amplifier.

The fetal heart sound was amplified three times in wireless fetal sound monitoring system: in the sensor, in the analog amplifier, and at the terminal. The amplification in the sensor ensures the weak fetal heart sound can be easily detected by the convector. The amplification at the terminal ensures the signal in the screen is easy to observe. Only the amplification in the amplifier amplifies the actual signal and represses the noise.

## 4. Adaptive Noise Reduction of the Fetal Heart Sound

When the fetal heart sound reached the sensor, it was mixed with the noise of the pregnant woman’s body so that the collected signal contained a lot of disturbance. The random noise was superposed to the low-frequency signal and burrs appeared in the signal curve. This is equal to a band whose center is the fetal heart sound signal and the width is the magnitude of the noise. The aim of noise reduction is to find the center line from the disturbed signal [8,9]. The adaptive noise reduction of the fetal heart sound was adopted to achieve this goal.

The training operation in the support vector regression (SVR) (Supplementary Materials) algorithm is relatively long, and the continual readjustment of C, the regular parameter, and γ, the bandwidth of the Gauss kernel, cannot satisfy real-time requirements. For this reason, the length of the trained data sample must be controlled. If it is too long, the time requirements cannot be met; if it is too short, the accuracy of C and γ cannot be satisfied. The fetal heart sound is periodic and the parameters can, in theory, only be determined in one period. The fetal heart rate varies from 120 to 160 beats per minute and two periods are contained in one second, so the data in one second were selected to train and to reduce noise. The fetal heart rate may change slowly and C and γ change with it. The threshold, θ, was set, and when the mean square error (MSE) was larger than θ, the current data were trained again to obtain an optimum C and γ. Cross-validation in deep-learning algorithms was used to monitor the status and prevent overfitting in training [10,11,12,13,14]. If MSE was smaller than θ, the current C and γ were regarded as perfect, the result was output, and the new data continued to reduce noise. The adaptive SVR noise reduction algorithm does not contain manually defined C and γ and obtains optimum parameters through self-learning according to the character of the real-time signal. Thus, the system adaptability was strengthened.

## 5. Extraction of Fetal Heart Rate

The direct calculation method or time-domain windowing can be used to extract the fetal heart rate [15,16]. The signal peak’s positions may drift and any tiny variation in denominator can be amplified. The direct calculation method outputs unsteadily and is not fit for real-time applications. Time-domain windowing was proposed to weaken the defect in the direct calculation method to some degree, as tiny changes in fetal heart rate are not uncommon [17]. If a spike is low that it is missed, the result will decrease by the smallest unit; meanwhile, if the disturbance is detected as a fetal heart sound peak, the result will increase by the smallest unit.

The weight associated with each peak interval causes the calculation of fetal heart rate to be incorrect in the condition of a missed or incorrect detection. Although the mean can weaken this disturbance, the calculation of fetal heart rate will be significantly influenced if the missed or incorrect detection is severe. The fetal heart sound data were weighted automatically in the window so that the noise was removed and the calculation accuracy of fetal heart rate was increased. The ability of the automatic weight algorithm to eliminate disturbance decreases when considerable noise is apparent in the window. In extreme cases, the noise may overwrite the data and cause the output to fluctuate greatly. It must be restrained so that the fetal heart rate value does not change greatly as compared to the previous interval. The weighted-index average method was adopted to modify the weight between windows. The weighted coefficients of each data point decreased exponentially with time, and the closer the data point, the larger the weighted coefficient. This can be shown as:

(2) $$\{\begin{array}{c} v_{0}=0,\\ v_{1}=\beta \cdot v_{0}+\left(1-\beta \right)\cdot \eta_{1}\\ v_{2}=\beta \cdot v_{1}+\left(1-\beta \right)\cdot \eta_{2}\\ \ldots \ldots \\ v_{t}=\beta \cdot v_{t-1}+\left(1-\beta \right)\cdot \eta_{t} \end{array}$$

where $v_{t}$ is the optimal value after weighted-index average at *t*, $\eta_{t}$ denotes the calculated fetal heart rate in the window at *t*, β is length factor between 0 and 1. $v_{t}$ is approximately equal to the mean of 1/(1−β) data:

(3) $$v_{t}=\left(1-\beta \right)\cdot \eta_{t}+\left(1-\beta \right)\cdot \beta^{2}\cdot \eta_{t-1}+\ldots +\left(1-\beta \right)\cdot \beta^{t}\cdot \eta_{0}$$

where:

(4) $${\left(1-\rho \right)}^{\frac{1}{\rho }}\approx \frac{1}{e}\approx 0.36$$

Let ρ=1−β, so:

(5) $${\left(\beta \right)}^{\frac{1}{1-\beta }}\approx \frac{1}{e}\approx 0.36$$

The influence factor fell to about a third after 1/(1−β) data. This had great influence on the result in the previous calculation because *v*_0_ initially equals zero. For this reason, the deviation should be modified and the iterative formula is:

(6) $$v_{t}=\frac{\beta \cdot v_{t-1}+\left(1-\beta \right)\cdot \eta_{t}}{1-\beta^{t}}$$

The numerator has important value when ‘t’ is small. It is demonstrated that $1-\beta^{t}$ is closing to 1 with the increment of t and the difference can be ignored after 1/(1−β) data. The output of the fetal heart rate becomes more stable after a precise calculation in the windows and weighted-index modification outside the windows. The proposed extraction algorithm of fetal heart rate is shown in Algorithm 1.
**Algorithm 1:** Proposed Fetal Heart Rate Extraction Algorithm
Input: *D*, fetal heart sound data with the time of *T* seconds, *H*, peak margin matrix, *F*0 = 0, *t* = 0, *w* = 4. 1. the fetal heart sound data were obtained from t to *t* + *w* in *D*; 2. sampling down, from 8 k to 1 k; 3. find out the position of *m* peaks; 4. calculate the interval of *m*−1 peak and sort them by size (largest to smallest) to obtain matrix *P*; 5. calculate the time difference of *m*−2 peak interval and obtain matrix *E*; 6. find out the index *i*, correspond to the minimum in *E*; 7. set E(i)=+∞, put *P*(*i*) and *P*(*i* + 1) into matrix *H*; 8. repeat step 5 and 6 until min(E(i))=∞; 9. calculate the mean of element in matrix *H*, *A_t_* = mean(*H*); 10. calculate the fetal heart rate in window: *f_t_* = 60/*A_t_*; 11. modify the fetal heart rate $F_{t}=\frac{\beta \cdot F_{t-1}+\left(1-\beta \right)\cdot f_{t}}{1-\beta^{t}}$; 12. move window backward: *t* = *t* + 1; 13. repeat 1–11 until *t* + *w* > *T*; Output: *F*_0_, *F*_1_, *L*, *F*_*t*−1_.

The amount of data was reduced through sampling down at step 2, so the time cost of the algorithm declined and real-time requirements were easy to achieve. The interval between adjacent peaks was calculated at step 4. The data were automatically weighted inside of the windows from step 3 to step 9. The fetal heart rate was modified by the weighted-index average method between windows from step 10 to step 13.

## 6. Experiment Result

### 6.1. Realization of the Collection Module

The fetal heart sound sensor was made of copper according to Figure 2, and the external disturbance was prevented by means of the airtight collection environment. The circular surface under the sensor was placed on the abdomen of the pregnant woman and all the energy, resulting from the enclosed air vibrating due to the fetal heart sound, was collected and sent to the PVDF film. Physical amplification occurs when the signal collected on the larger inductive surface is applied to the relatively small film. The energy loss is negligible inside the enclosed cavity and the weak fetal heart sound can be detected and collected easily. The external electromagnetic environment is complex and copper cover can block the interference in a great degree.

**Figure 4:**
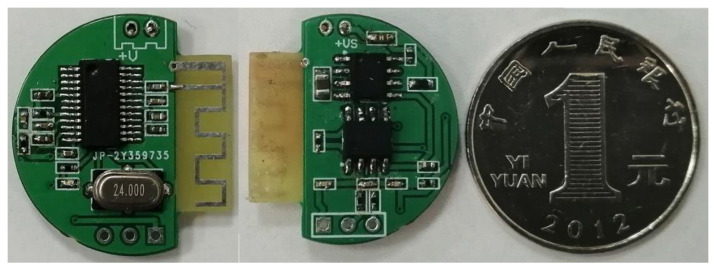
Collection circuit of the portable wireless fetal sound monitoring system.

The portable wireless fetal sound monitoring system’s collection circuit was made up of a two-layer printed circuit board (PCB) and is shown in Figure 4.

The circuit was minimized, its power was reduced, saving space and improving functionality. The PCB was designed as a circular plate in order to fit the sensor and reduce the general size. The left image in the Figure 4 is the front, which is responsible for the transmission of the wireless signal. The right image is the back, which is for power management, signal input, and amplification.

### 6.2. Experiment of Adaptive Noise Reduction

**Figure 5:**
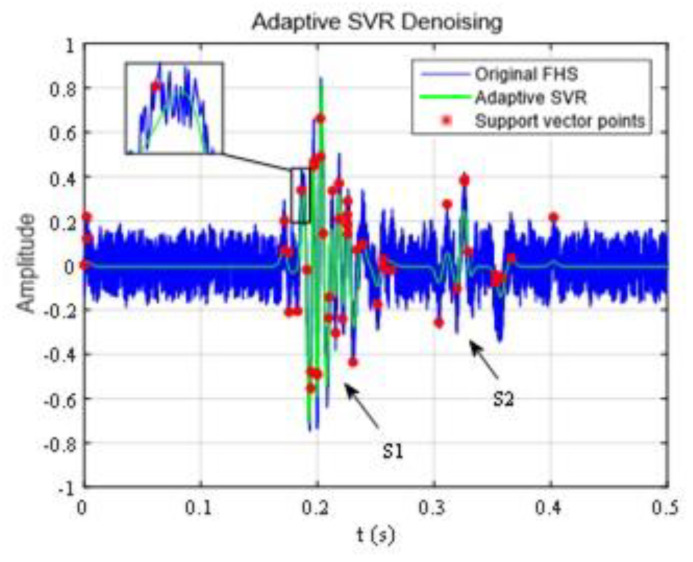
Adaptive Support Vector Regress (SVR) noise reduction of fetal heart sound.

The fetal heart sound data were cut into 0.5 s, ε=0.1, θ=0.1. The magnitude was normalized and the signal after noise reduction is shown in Figure 5.

The noise in the original fetal heart sound data is larger and the second heart sound is almost completely covered. The adaptive SVR algorithm followed well in the first and second heart sounds and inhibited the noise effectively in the other part. The tendency of the fitting curve was severely restricted by the points of the SVR.

**Figure 6:**
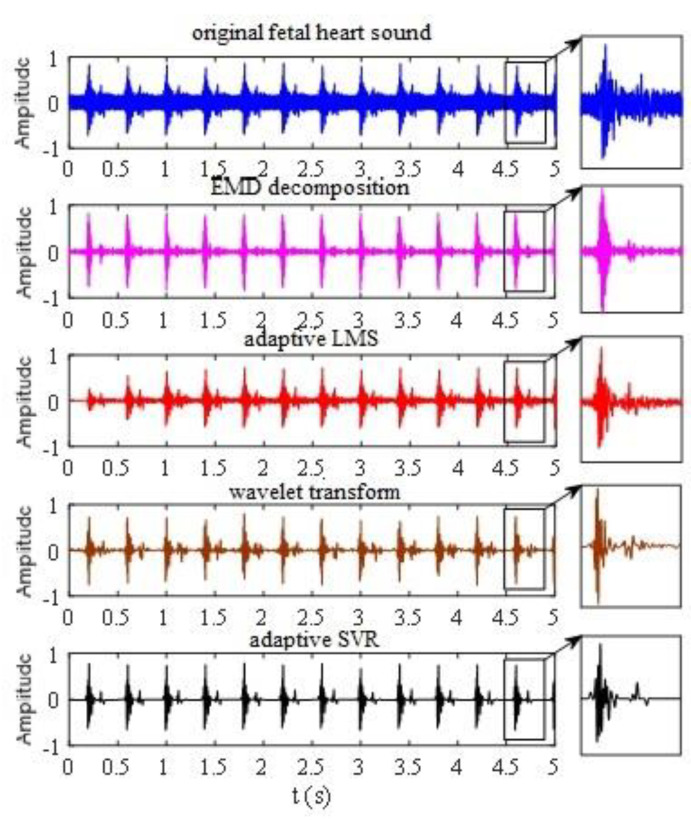
Comparison of noise reduction of the four algorithms.

The factorization algorithm with EMD, the self-adaptive least squares method (LSM), and the wavelet transform algorithm were employed to reduce noise in the original fetal heart sound and were compared with the de-noised results of the adaptive SVR algorithm [18,19]. In the factorization algorithm with EMD, the factorization layer was 10 and the correlation calculation was operated between the implicit matrix factorization (IMF) component after factorization and the original signal. The signal was reconstructed using half the IMF component with a large correlation coefficient. The length of the filter was 51 in the self-adaptive LSM and the learning rate was 0.0014. The wavelet basis functions in the wavelet transform algorithm were sym5 and the factorization layer was 6. The threshold was obtained using minimax methods. The coefficient was processed using the hard-threshold function and the signal was reconstructed to reduce the noise. The noise reduction results of these algorithms are shown in Figure 6.

These four algorithms all reduced noise to some degree. The signal-to-noise ratios (SNR) of EMD decomposition, adaptive LMS, wavelet transform, and adaptive SVR were 10.36, 14.42, 20.64, and 22.17, respectively. The first fetal heart sound could be separated from the original by the factorization algorithm with EMD. However, the details of the second fetal heart sound were lost because of noise floor, and the noise requirement cannot be satisfied [20]. Compared with factorization algorithm with EMD, self-adaptive LSM retained part of the second fetal heart sound and reduced the noise.

Both the wavelet transform algorithm and the adaptive SVR algorithm performed well and were superior to the others. These two methods followed the first the second fetal heart sounds well. However, for the low noise, the adaptive SVR algorithm performed much better than the wavelet transform algorithm. The wavelet transform algorithm fluctuated without noise, while a straight line was obtained by the adaptive SVR algorithm. Furthermore, the wavelet basis function had to be selected and the hierarchy determined in the wavelet transform, and this has a great influence on noise reduction. Although similar parameters, C and γ, should be set in the SVR algorithm, their optimal value can be obtained through self-learning by adaptive means and can be adjusted according to the characteristics of the signal.

### 6.3. Fetal Heart Rate Extraction Experiment

**Figure 7:**
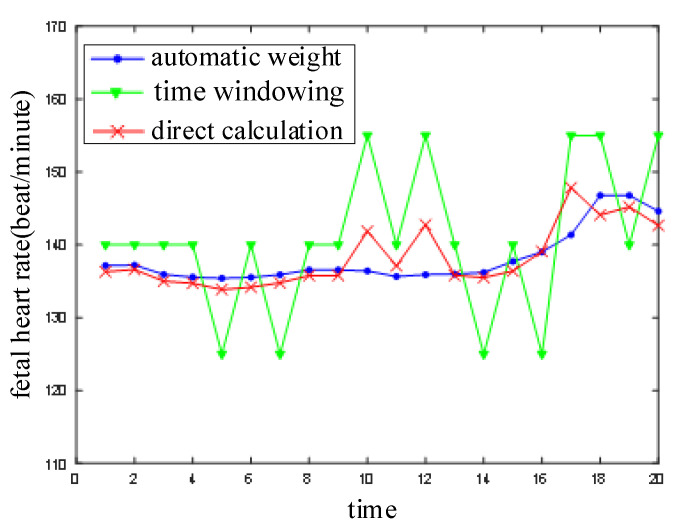
Comparison of three fetal heart rate extraction algorithms.

Firstly, three fetal heart rate extraction algorithms were compared in one window. Then, the actual results, which were modified by the weighted-index average algorithm between windows, were inspected. Twenty fetal heart rate data points of the same group were calculated continuously using three independent algorithms. The time of the window was 4 s and it moved forward 1 s at a time. The results are shown in Figure 7.

In order to homogenize the result dimensions, the average of the peak intervals in the windows was adopted when the fetal heart rate was directly calculated. It can be seen that the minimum variation of fetal heart rate is 15 with the time-domain windowing because there are 15 windows in 1 min. The effect of time-domain windowing is poor for the short time window. This may be due to that fact that three disturbances occurred in twenty data points. Both the direct calculation method and the automatic weight method performed well when the noise appeared for the first time. The influence of the noise was weakened by the average effect used in the direct calculation algorithm. However, when the noise appeared for the second time, it was not completely eliminated, although it was weakened on average. The average effects will decrease with excessive noise and over longer times. The disturbance was eliminated automatically so that the automatic weight algorithm results were more stable.

**Figure 8:**
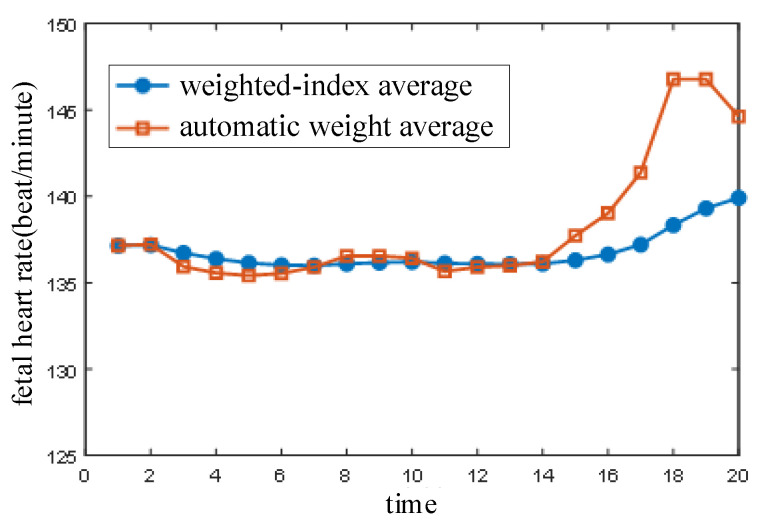
Weighted-index average algorithm.

The automatic weight algorithm has defects and it cannot satisfy the demands of a real-time system, although it improved the performance to some degree as compared with the other two algorithms. For example, the fetal heart rate rose too quickly, which lead to a jump from the sixteenth to the eighteenth calculation. In general, the fetal heart rate varies slowly and does not jump in a short time. The jumping was caused by disturbance. The fetal heart rate data obtained using the weighted-index average method with deviation correction were further processed on the basis of this fetal heart rate characteristic. The length factor, β=0.9, and the result are shown in Figure 8.

The fetal heart rate was corrected by the weighted-index average algorithm on the basis of extraction using the automatic weight algorithm. The subtle fluctuation was eliminated from the second calculation to the twelfth and the fetal heart rate tended to rise from the fourteenth to the twentieth. The jumping fetal heart rate was weakened by the weighted-index average algorithm and the variation in the fetal heart rate became more stable. The expected effect was realized and the results were consistent with reality.

## 7. Conclusions

A wireless high-sensitivity fetal heart sound monitoring system is composed of two parts: fetal heart sound collection and fetal heart sound processing. The reported exclusive sensor, which was constructed of copper with an enclosed cavity, was designed to prevent EMI and external noise. PVDF piezoelectric film, with a frequency from DC to 400 MHz, was adopted to convert the fetal heart sound into an electrical signal without information loss. The collected fetal heart sound was amplified and transmitted to the terminal wirelessly. The adaptive SVR algorithm, which optimizes parameters according the characteristics of the real-time system and ensures output stability, was proposed to reduce noise. Fetal heart rate extraction includes operation inside and between windows. The automatic weight algorithm was proposed to eliminate missed or incorrect detections in the windows and was more stable than the two known algorithms. The weighted-index average algorithm with deviation correction was processed to restrain the calculated fetal heart rate according to the continuity in time. The wireless fetal heart sound monitoring system can process fetal heart sound correctly and in real time.

## Data Availability

Data sharing not applicable.

## Supplementary Materials

The following are available online at https://www.mdpi.com/1424-8220/21/1/193/s1, Figure S1: Adaptive Support Vector Regress (SVR) noise reduction algorithm.

## References

[1] Hamelmann P., Mischi M., Vullings R., Kolen A.F., Schmitt L., Joshi S., van Laar J.O.E.H., Bergmans J.W.M. Flexible sensor matrix with dynamic channel weighting for improved estimation of the fetal heart rate by doppler ultrasound. Proceedings of the 2017 IEEE International Ultrasonics Symposium (IUS).

[2] Khandoker A.H., Alangari H.M., Marzbanrad F., Kimura Y. Investigating fetal myocardial function in heart anomalies by Doppler myocardial performance indices. Proceedings of the 39th Annual International Conference of the IEEE Engineering in Medicine and Biology Society (EMBC).

[3] Sato H., Yoshimura K., Nakamoto H., Ishibashi D., Nakata Y., Yaginuma Y., Masui S. 19.2 cm^3^ flexible fetal heart rate sensor for improved quality of pregnancy life. Proceedings of the 2016 IEEE Biomedical Circuits and Systems Conference (BioCAS).

[4] Andreotti F., Grasser F., Malberg H., Zaunseder S. (2017). Non-Invasive Fetal ECG Signal Quality Assessment for Multichannel Heart Rate Estimation. IEEE Trans. Biomed. Eng..

[5] Kovacs F., Goda M.A., Hosszu G., Telek T. A Proposed Phonography-Based Measurement of Fetal Breathing Movement Using Segmented Structures with Frequency Splitting. Proceedings of the 2020 42nd Annual International Conference of the IEEE Engineering in Medicine & Biology Society (EMBC).

[6] Lakshmi G.S., Bhaskaran A., Bharadwaj S.U., Arora M. Effect of Contact Force on Foetal Heart Sound Recordings. Proceedings of the 2020 IEEE International Conference on Electronics, Computing and Communication Technologies (CONECCT).

[7] Eiríksdóttir D.H., Sæderup R.G., Riknagel D., Zimmermann H., Plocharski M., Hansen J., Struijk J.J., Schmidt S.E. Quality Assessment of Maternal and Fetal Cardiovascular Sounds Recorded from the Skin Near the Uterine Arteries during Pregnancy. Proceedings of the 2019 Computing in Cardiology (CinC).

[8] Shengwei W., Yanni L., Jiayu Z., Jiajia L. Agricultural price fluctuation model based on SVR. Proceedings of the 9th International Conference on Modelling, Identification and Control (ICMIC).

[9] Mesquita D.P., Freitas L.A., Gomes J.P., Mattos C.L. (2020). LS-SVR as a Bayesian RBF Network. IEEE Trans. Neural Netw. Learn. Syst..

[10] Ni L., Li J., Lin S., Xin D. A method of noise optimization for Hardware Trojans detection based on BP neural network. Proceedings of the 2nd IEEE International Conference on Computer and Communications (ICCC).

[11] Isogawa K., Ida T., Shiodera T., Takeguchi T. (2018). Deep Shrinkage Convolutional Neural Network for Adaptive Noise Reduction. IEEE Signal Process. Lett..

[12] Wu C., Ma J., Zhang X. (2020). User space transformation in deep learning based recommendation. J. Syst. Eng. Electron..

[13] Daryanavard S., Porr B. (2020). Closed-Loop Deep Learning: Generating Forward Models with Backpropagation. Neural Comput..

[14] Zhu H., Wang W., Leung R. (2020). SAR Target Classification Based on Radar Image Luminance Analysis by Deep Learning. IEEE Sens. Lett..

[15] Romano M., Iuppariello L., D’Addio G., Clemente F., Amato F., Cesarelli M. Computerised simulation of fetal heart rate signals. Proceedings of the 2017 E-Health and Bioengineering Conference (EHB).

[16] Fuentealba P., Illanes A., Ortmeier F. (2019). Cardiotocographic Signal Feature Extraction through CEEMDAN and Time-Varying Autoregressive Spectral-Based Analysis for Fetal Welfare Assessment. IEEE Access.

[17] Gurun G., Zahorian J.S., Sisman A., Karaman M., Hasler P.E., Degertekin F.L. (2012). An Analog Integrated Circuit Beamformer for High-Frequency Medical Ultrasound Imaging. IEEE Trans. Biomed. Circuits Syst..

[18] Sbrollini A., Strazza A., Caragiuli M., Mozzoni C., Tomassini S., Agostinelli A., Morettini M., Fioretti S., Di Nardo F., Burattini L. Fetal Phonocardiogram Denoising by Wavelet Transformation: Robustness to Noise. Proceedings of the 2017 Computing in Cardiology (CinC).

[19] Kahankova R., Martinek R. Comparison of Fetal Phonocardiogram Wavelet Denoising Methods. Proceedings of the 2018 IEEE Signal Processing in Medicine and Biology Symposium (SPMB).

[20] Wang Z., Wei J., Li X. Adaptive SVR Denoising Algorithm for Fetal Monitoring System. Proceedings of the 2018 10th International Conference on Wireless Communications and Signal Processing (WCSP).

