# Supplementary material for: A Wireless High-Sensitivity Fetal Heart Sound Monitoring System
Source: Sensors (Basel). 2020 Dec 30;21(1):193. doi: 10.3390/s21010193 (PMC7794984; doi:10.3390/s21010193)
Supplement: Supplementary file 1 [file sensors-21-00193-s001.pdf]

Supplementary Section

In the Support Vector Regress Algorithm, the cost function is

$$Cost = \sum_{i=1}^{m} l_\varepsilon(f(x_i) - y_i) \quad \text{(s.1)}$$

where (x_i,y_i) are the training samples, f(x_i) is the target regression equation, l_ε is the insensitive loss function to ε, which can be represented as

$$l_\varepsilon(z) = max(0, |z| - \varepsilon) \quad \text{(s.2)}$$

The regularization term l_2-norm was adopted to restrict large weights and to reduce the dimensionality of fitting curves so that the problem of overfitting was slowed down. Therefore, the problem of SVR was converted into

$$\min_{w,b} \frac{1}{2}\|w\|^2 + C\sum_{i=1}^{m} \ell_\varepsilon(f(x_i) - y_i) \quad \text{(s.3)}$$

where $w^T x + b = 0$ is hyperplane, C is the regular parameter, which was used to control the weight of the regular term in the whole cost function and represented the emphasis to the outlier. In order to obtain optimal regularization constants through data training, the relaxation variable, ξ_i和ξ_i^*, was introduced and Equation(s.3) was converted to

$$\begin{aligned} &\min_{w,b,\xi,\xi^*} \frac{1}{2}\|w\|^2 + C\sum_{i=1}^{m}(\xi + \xi^*) \\ &s.t.\ -(\varepsilon + \xi^*) \le f(x_i) - y_i \le \varepsilon + \xi \quad \text{(s.4)} \\ &\xi^* \ge 0, \xi^* \ge 0,\ i=1,2,\dots,m \end{aligned}$$

The duality of Equation (s.4) can be obtained through the Lagrange multiplier method:

$$\begin{aligned} \max_{\alpha,\alpha^*} \quad & \sum_{i=1}^{m} y_i(\alpha_i^* - \alpha_i) - \varepsilon(\alpha_i^* + \alpha_i) \\ & -\frac{1}{2}\sum_{i=1}^{m}\sum_{j=1}^{m}(\alpha_i^* - \alpha_i)(\alpha_j^* - \alpha_j)x_i^T x_j \quad \text{(s.5)} \\ s.t. \quad & \sum_{i=1}^{m}(\alpha_i^* - \alpha_i) = 0, \\ & 0 \le \alpha_i, \alpha_i^* \le C. \end{aligned}$$

The requirement of KKT(Karush–Kuhn–Tucker) must be met:

$$\begin{cases} a_i(f(x_i) - y_i - \varepsilon - \xi_i) = 0, \\ a_i^*(y_i - f(x_i) - \varepsilon - \xi_i^*) = 0, \\ a_i a_i^* = 0, \xi_i \xi_i^* = 0, \\ (C - a_i)\xi_i = 0, (C - a_i^8)\xi_i^* = 0 \end{cases} \quad \text{(s.6)}$$

From Equation (s.6), it can be deduced that one of the two items a_i or $(f(x_i) - y_i - \varepsilon - \xi_i)$ must be at least zero. For the same reason, a_i^* and $(y_i - f(x_i) - \varepsilon - \xi_i^*)$ also satisfy this demand. When the samples fall into an acceptable region, both a_i and a_i^* are zero and are not involved in error calculation. On the contrary, in an unacceptable region, a_i and a_i^* maybe nonzero and should be used to calculate loss. Apart from this, either $(f(x_i) - y_i - \varepsilon - \xi_i)$ or $(y_i - f(x_i) - \varepsilon - \xi_i^*)$ must be nonzero at the same time and a_i and a_i^* must be at least zero. The solution of SVR is

$$f(x) = \sum_{i=1}^{m}(a_i^* - a_i)x_i^T x + b \quad \text{(s.7)}$$

The kernel was introduced based on the SVR primitive equation and equation (s.7) was converted into

$$f(x) = \sum_{i=1}^{m}(a_i^* - a_i)k(x, x_i) + b \quad \text{(s.8)}$$

The Gauss kernel, $k(x_i, x_j) = exp\left(-\gamma\|x_i - x_j\|^2\right)$, was adopted, where γ>0 was the bandwidth of Gauss kernel.

In Equation (s.8), only the sample at $a_i^* - a_i \neq 0$ are the support vector of SVR outside of the ε margin. Both a_i^* and a_i are zero inside, and only part samples were used by SVR. The data, (x,y), were trained to deduce C and γ before the SVR algorithm was adopted to reduce noise. The signal was modified by itself and the adaptive noise reduction was realized. The algorithm is illustrated in supplementary Figure S1. 'Flag' is the control flag and is set by the main program.

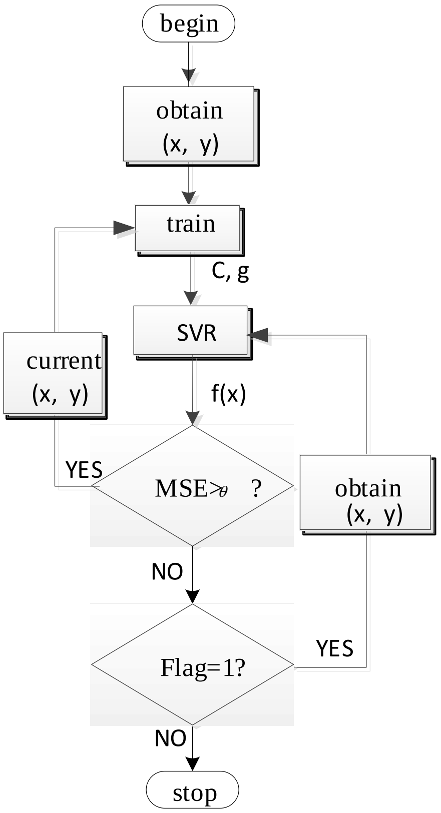

Figure S1. Adaptive Support Vector Regress (SVR) noise reduction algorithm.
